# Collateral estimation by susceptibility-weighted imaging and prediction of functional outcomes after acute anterior circulation ischemic stroke

**DOI:** 10.1038/s41598-021-00775-9

**Published:** 2021-11-01

**Authors:** Hyung Jin Lee, Hong Gee Roh, Sang Bong Lee, Yoo Sung Jeon, Jeong Jin Park, Taek-Jun Lee, Yu Jin Jung, Jin Woo Choi, Young Il Chun, Hee Jong Ki, Junsoo Cho, Ji Sung Lee, Hyun Jeong Kim

**Affiliations:** 1 Department of Neurosurgery, Daejeon St. Mary’s Hospital, College of Medicine, The Catholic University of Korea, Daejeon, Korea; 2 Department of Radiology, Konkuk University Medical Center, Seoul, Korea; 3 Department of Neurology, Daejeon St. Mary’s Hospital, College of Medicine, The Catholic University of Korea, Daejeon, Korea; 4 Department of Neurosurgery, Konkuk University Medical Center, Seoul, Korea; 5 Department of Neurology, Konkuk University Medical Center, Seoul, Korea; 6 Clinical Research Center, Asan Institute for Life Science, Asan Medical Center, University of Ulsan College of Medicine, Seoul, Korea; 7 Department of Radiology, Daejeon St. Mary’s Hospital, The Catholic University of Korea, 64 Daeheung-Ro, Jung-Gu, Daejeon, 34943 Korea

**Keywords:** Biomarkers, Diseases, Medical research, Neurology

## Abstract

To determine the value of susceptibility-weighted imaging (SWI) for collateral estimation and for predicting functional outcomes after acute ischemic stroke. To identify independent predictors of favorable functional outcomes, age, sex, risk factors, baseline National Institutes of Health Stroke Scale (NIHSS) score, baseline diffusion-weighted imaging (DWI) lesion volume, site of steno-occlusion, SWI collateral grade, mode of treatment, and successful reperfusion were evaluated by multiple logistic regression analyses. A total of 152 participants were evaluated. A younger age (adjusted odds ratio (aOR), 0.42; 95% confidence interval (CI) 0.34 to 0.77; P < 0.001), a lower baseline NIHSS score (aOR 0.90; 95% CI 0.82 to 0.98; *P* = 0.02), a smaller baseline DWI lesion volume (aOR 0.83; 95% CI 0.73 to 0.96; *P* = 0.01), an intermediate collateral grade (aOR 9.49; 95% CI 1.36 to 66.38; *P* = 0.02), a good collateral grade (aOR 6.22; 95% CI 1.16 to 33.24; *P* = 0.03), and successful reperfusion (aOR 5.84; 95% CI 2.08 to 16.42; *P* = 0.001) were independently associated with a favorable functional outcome. There was a linear association between the SWI collateral grades and functional outcome (*P* = 0.008). Collateral estimation using the prominent vessel sign on SWI is clinically reliable, as it has prognostic value.

## Introduction

Acute ischemic stroke is a leading cause of death and major disability worldwide^1^. Intravenous thrombolysis (IVT) with tissue plasminogen activator and intraarterial thrombectomy (IAT) has revolutionized the treatment of acute ischemic stroke and been shown to be effective in improving the functional outcome of patients with acute ischemic stroke within 4.5 h and 6 h after symptom onset, respectively^2–8^. Time is an important factor in the effectiveness of IVT and IAT since ischemia evolves over time after acute stroke^9,10^. However, the treatment effect was not constant even in the optimal time window. Futile endovascular treatment ranged from 29 to 67% in the recent randomized clinical trials^4–8^ and hemorrhagic transformation worsen the functional outcome ranged from 2.4 to 8.8%^2,3,11–14^. Recent studies have shown that endovascular treatment could be effective in select patients up to 24 h after symptom onset^15,16^. These differences in the treatment effect beyond the time window are due to individual differences in the pace of infarct progression by collateral circulation. Collaterals are alternative vascular channels for maintaining blood perfusion to the ischemic brain distal to an arterial occlusion; therefore, infarct progression depends on the collateral status, which varies among patients^17^. Infarction may occur within less than 1 h or may last for hours or days^18,19^. Recanalization and reperfusion treatment can be futile or even dangerous when performed in the optimal time window but could be useful if performed at a later time. Meta-analyses showed that better collateral circulation may lead to favorable functional outcome, better recanalization or reperfusion rates, a smaller infarct core, a lower hemorrhagic transformation rate and a lower risk of mortality after IVT and IAT for various durations in patients with acute ischemic stroke^20,21^. Collateral estimation is being considered increasingly important to precisely select eligible patients for IVT and IAT. Recently, useful collateral imaging methods using contrast material have been introduced, and they have shown prognostic value in acute cerebral ischemia due to large-vessel occlusion^22–25^. However, a noncontrast imaging method for collateral estimation is necessary with consideration of contrast-induced complications which can lead to irreversible kidney damage and an increased risk of morbidity and mortality, especially for patients with chronic kidney disease, which is a major risk factor for postcontrast acute kidney injury^26–28^.

Susceptibility-weighted magnetic resonance imaging (SWI) is gradient recalled echo T2*-weighted imaging. SWI is an essential sequence in the baseline magnetic resonance imaging protocol used for evaluating patients with acute cerebral ischemia due to its high sensitivity to deoxygenated hemoglobin in the brain and cerebral vessels. In addition to the clinical significance of hemorrhage detection^29^, studies have shown that the prominent vessel sign (prominent cortical and/or medullary vein) of ischemic brain on SWI can be considered an imaging biomarker representing collateral status and a predictor of functional outcomes^30–35^. The prominent vessel sign has been positively correlated with the amount of deoxyhemoglobin in cerebral veins secondary to an increased oxygen extraction fraction of the ischemic brain, which shortens the T2* relaxation and decreases the signal intensity of the veins in the ischemic region with respect to that in a normal brain^36^. Therefore, it can be hypothesized that ischemic tissue with poor collaterals requires more oxygen and contains more deoxyhemoglobins in tissue vessels, demonstrating a more prominent vessel sign on SWI than that of tissue with good collaterals. However, the results regarding the correlations between the prominent vessel sign and collateral status and the prognosis of ischemic stroke are inconsistent. Verma et al. showed that extensive prominent cortical veins correlated with poor leptomeningeal collateralization, while less pronounced prominent cortical veins correlated with good leptomeningeal collateralization^30^; however, Park et al. showed that more extensive prominent vessels were associated with better collateral flow^37^.

Validation of the role of prominent vessel signs on SWI as a collateral marker can enable collateral estimation without the use of contrast material or additional imaging. This method is particularly useful in patients who are unable to undergo contrast-based radiological studies. This study was conducted to verify the value of prominent vessel signs for estimating collateral status and for predicting functional outcomes in patients with acute ischemic stroke due to large-vessel occlusion in the anterior circulation.

## Methods

The local institutional review boards of Konkuk University Medical Center (KUMC0114-01-100-007) and Daejeon St. Mary’s Hospital (DC16OIMI0043) approved this prospective observational study, and written informed consent was obtained from all participants. All methods were carried out in accordance with relevant guidelines and regulations of the Declaration of Helsinki and the Strengthening the Reporting of Observational Studies in Epidemiology (STROBE) guidelines for reporting observational study.

### Study participants

This secondary analysis used published data obtained from a prospective observational study in patients with acute ischemic stroke due to occlusion or stenosis of the unilateral internal carotid artery (ICA) and/or M1 segment of the middle cerebral artery (MCA) who were evaluated within 8 h of symptom onset. The details of the study population and imaging methods have been described previously^24,25^. Participants were evaluated for demographic data, medical history, vascular risk factors, routine blood tests, brain imaging, and cardiological tests. The stroke severity was assessed with the National Institutes of Health Stroke Scale (NIHSS). Participants underwent one of the following treatments at the discretion of the attending physician: intravenous thrombolysis, intraarterial thrombectomy, intravenous thrombolysis followed by intraarterial thrombectomy, or conservative treatment. Recanalization and reperfusion were assessed on the basis of conventional cerebral angiograms obtained at the end of the intra-arterial procedure and were classified by using the modified thrombolysis in cerebral infarction, or mTICI, system as follows: 0, no reperfusion; 1, penetration with minimal reperfusion; 2a, < 50% reperfusion; 2b, ≥ 50% reperfusion; 2c, near-complete reperfusion; and 3, complete reperfusion of the affected vascular territory. Reperfusion status was classified as either successful reperfusion (mTICI score of 2b to 3) or failed reperfusion (mTICI score of 0 to 2a). The participants who did not undergo endovascular treatment were also classified as having failed reperfusion. The functional outcomes were assessed on day 90 with the modified Rankin scale; a favorable functional outcome was defined as a modified Rankin scale score of 2 or less on day 90.

### Imaging protocol and postprocessing

All participants underwent MRI, including diffusion-weighted imaging (DWI), SWI, arterial spin labeling, dynamic contrast-enhanced MR angiography, dynamic susceptibility contrast-enhanced MR perfusion and fluid attenuation inversion recovery (FLAIR). MRI was performed with 3-Tesla MRI scanners (Skyra, Siemens Healthcare, Erlangen, Germany and Ingenia, Philips Healthcare, Best, Netherlands). The acquisition parameters were the same as those used in a previous study^24^. An experienced neuroradiologist (J.W.C with 14 years of experience), who was blinded to all clinical and other imaging data, measured the infarct volume (in milliliters) by using Medical Image Processing, Analysis, and Visualization (MIPAV, version 7.1.1; National Institutes of Health, Bethesda, MD)^25^. By using source data from dynamic contrast-enhanced MR angiography, 3-dimensional rotational arteriography was performed to determine the arterial status, and a multiphase MR angiography collateral map was reconstructed to evaluate the collateral-perfusion status. We generated the multiphase MR angiography collateral map using a MATLAB-based in-house program (version 9.6.0, MathWorks, Natick, MA)^24^.

### Imaging analysis

**Figure 1:**
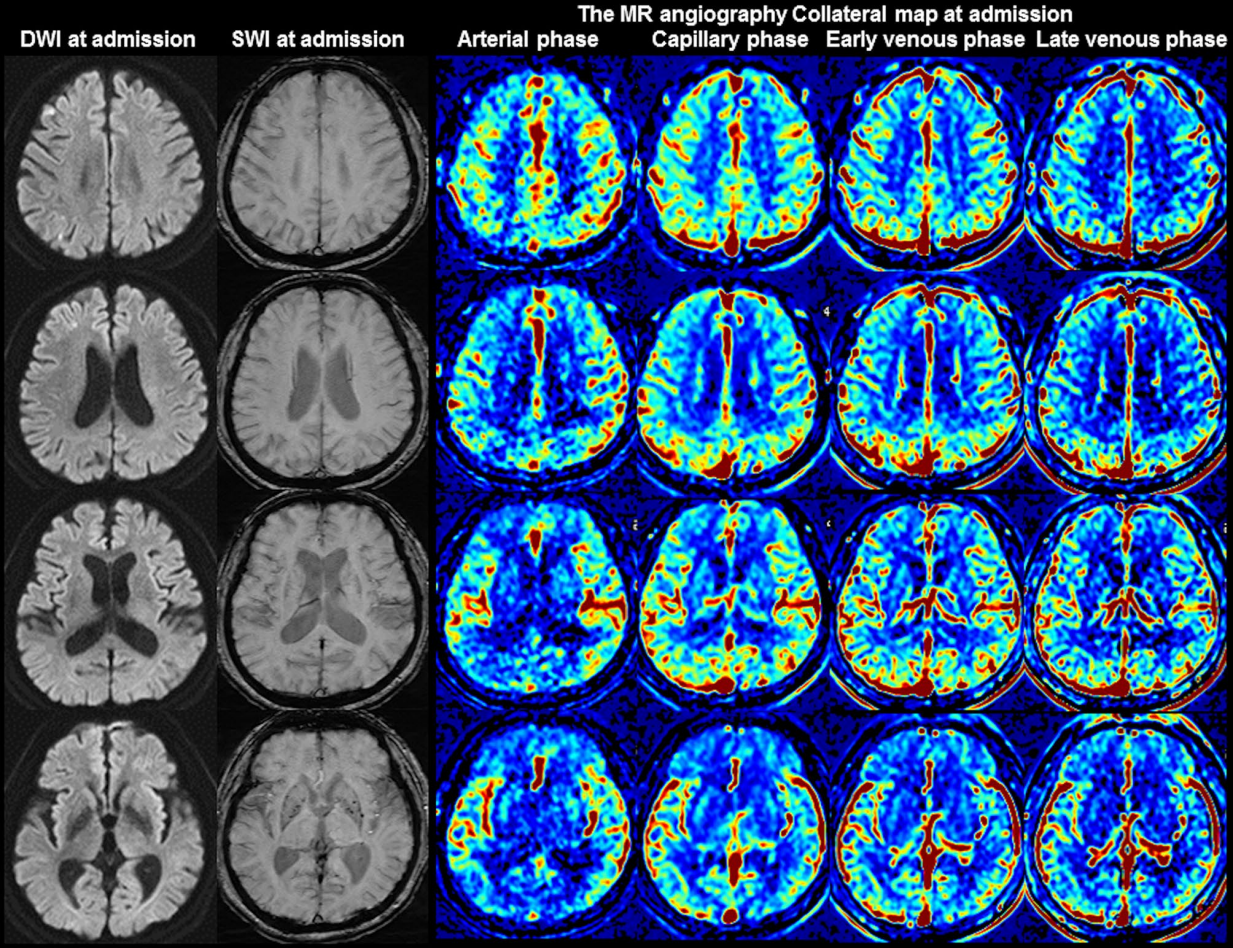
Images of a 68-year-old man with greater than 70% stenosis of the right proximal internal carotid artery. The premorbid modified Rankin scale (mRS) score of this patient was 0, and the National Institutes of Health Stroke Scale score at admission was 1. The diffusion-weighted imaging (DWI), susceptibility-weighted imaging (SWI), and multiphase MR angiography collateral map at 44 min after symptom onset are shown. DWI shows acute infarct signals along the border zones of the right cerebral hemisphere, and SWI shows no prominent cortical and medullary veins in the right cerebral hemisphere, representing good collateral status. The MR angiography collateral map shows no collateral-perfusion delay in the right cerebral hemisphere in the capillary phase (MR acute ischemic stroke collateral score of 5: excellent collateral perfusion defined as no or small collateral-perfusion delay in the ischemic MCA territory in the capillary phase regardless of the collateral status in the arterial phase). The patient underwent conservative treatment and recovered, as shown by the 90-day mRS score of 0.

**Figure 2:**
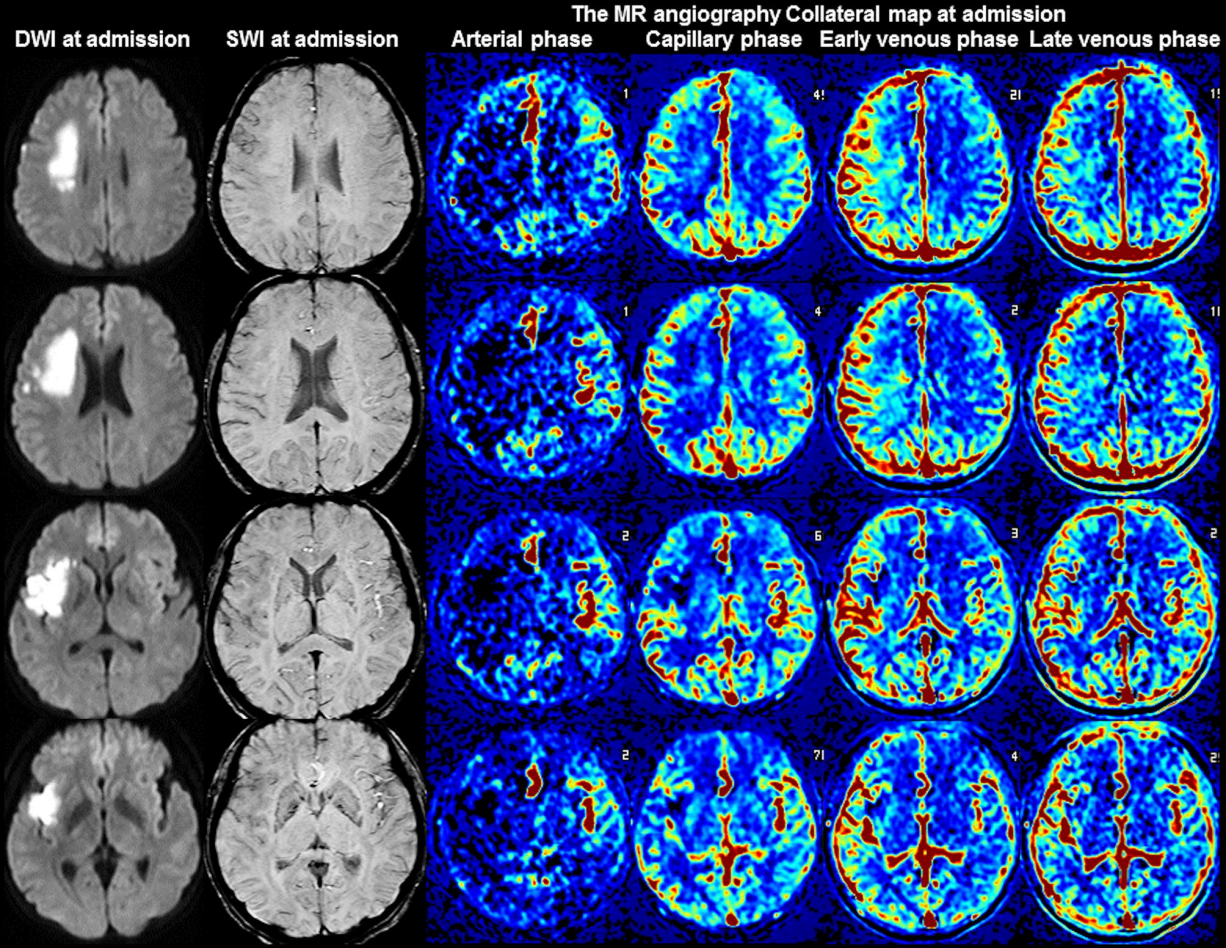
Images of a 55-year-old woman with occlusion of the right proximal internal carotid artery. The premorbid modified Rankin scale (mRS) score of this patient was 0, and the National Institutes of Health Stroke Scale score at admission was 4. The diffusion-weighted imaging (DWI), susceptibility-weighted imaging (SWI), and multiphase MR angiography collateral map at 4 h 45 min after symptom onset are shown. DWI shows acute infarct signals in the right frontal and temporal lobes, and SWI shows mildly prominent cortical veins in the right middle cerebral artery territory, representing intermediate collateral status. The MR angiography collateral map shows collateral-perfusion delay of more than one-half of the right middle cerebral artery territory in the capillary phase and small collateral-perfusion delay around the insula in the early venous phase (MR acute ischemic stroke collateral score of 3: intermediate to good collateral perfusion defined as capillary-perfusion delay of more than one-half of the ischemic MCA territory in the capillary phase and no or small delay in the early venous phase). The patient underwent bypass surgery and recovered, as shown by the 90-day mRS score of 2.

**Figure 3:**
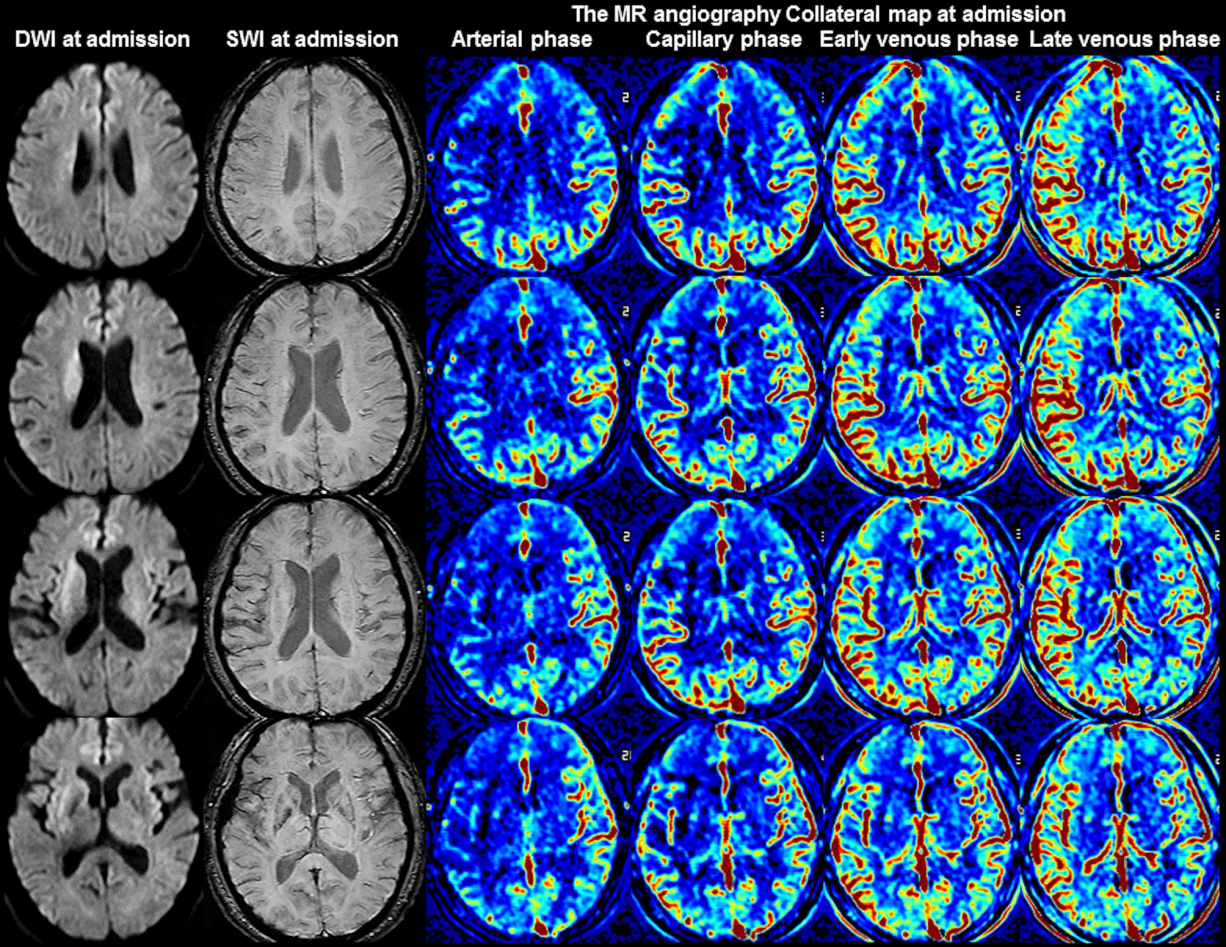
Images of a 62-year-old woman with occlusion of the right middle cerebral artery. The premorbid modified Rankin scale (mRS) score of this patient was 0, and the National Institutes of Health Stroke Scale score at admission was 8. The diffusion-weighted imaging (DWI), susceptibility-weighted imaging (SWI), and multiphase MR angiography collateral map at 1 h 40 min after symptom onset are shown. DWI shows acute infarct signals in the right basal ganglia, and SWI shows moderately prominent cortical and medullary veins in the right middle cerebral artery territory, representing poor collateral status. The MR angiography collateral map shows collateral-perfusion delay of more than one-half of the right middle cerebral artery territory in the capillary phase and less than one-half of the territory in the early venous phase (MR acute ischemic stroke collateral score of 2: intermediate to poor collateral perfusion defined as collateral-perfusion delay more than one-half of the ischemic MCA territory in the capillary phase and equal to or less than one-half in the early venous phase). The patient underwent intravenous thrombolysis followed by intraarterial thrombectomy, and the occluded arteries were completely recanalized at 2 h 45 min after symptom onset. The patient recovered, as shown by the 90-day mRS score of 1.

**Figure 4:**
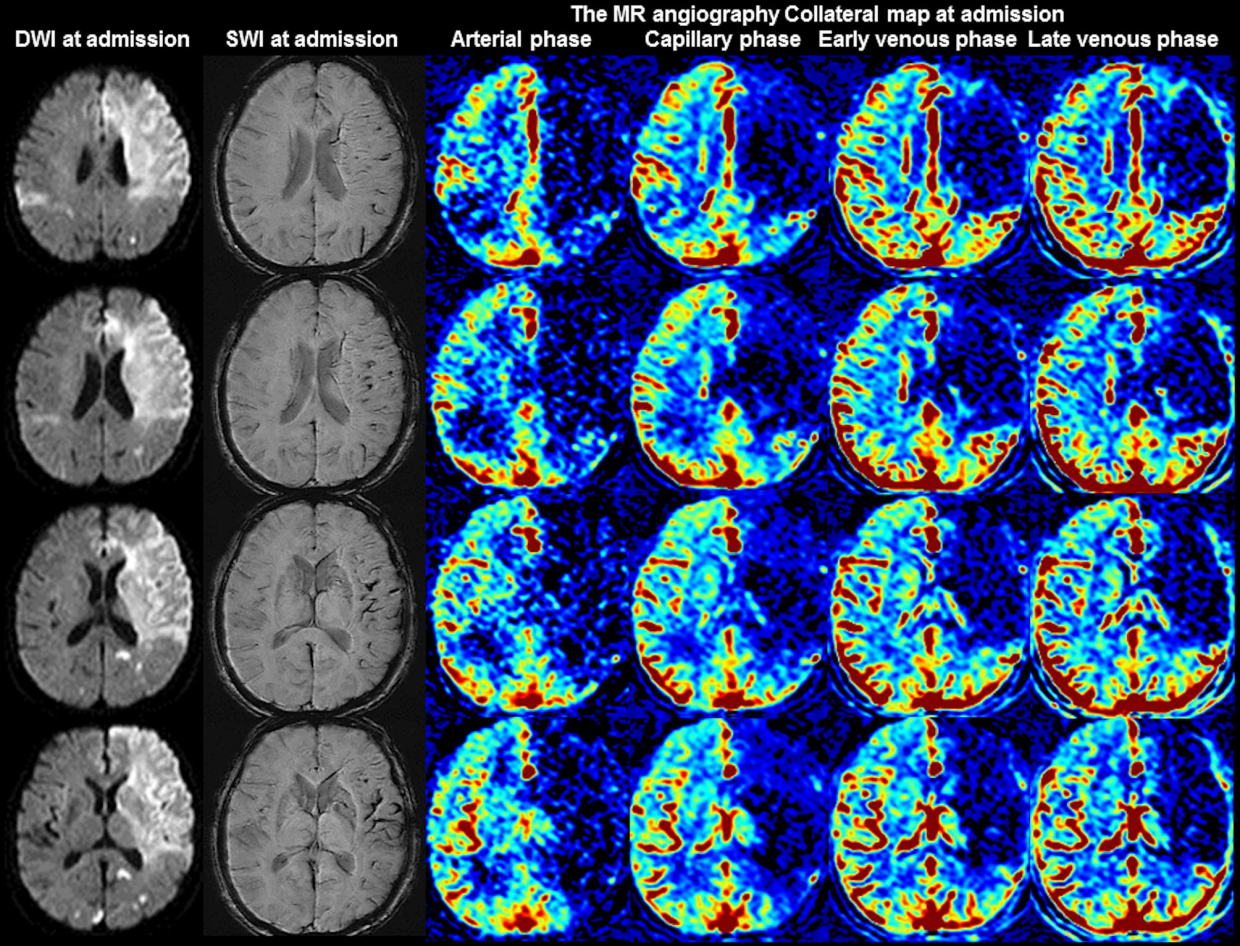
Images of a 72-year-old woman with occlusion of the left internal carotid and middle cerebral arteries. The premorbid modified Rankin scale (mRS) score of this patient was 0, and the National Institutes of Health Stroke Scale score at admission was 20. The diffusion-weighted imaging (DWI), susceptibility-weighted imaging (SWI), and multiphase MR angiography collateral map at 5 h and 25 min after symptom onset are shown. DWI shows acute infarct signals in both cerebral hemispheres. Acute infarction in the right cerebral hemisphere was considered to indicate another embolic infarction. SWI shows very prominent cortical and medullary veins in the left middle cerebral artery territory, representing very poor collateral status. The collateral-perfusion delay of more than one-half of the left middle cerebral artery territory persisted until the late venous phase on the MR angiography collateral map (MR acute ischemic stroke collateral score of 0: very poor collateral perfusion defined as collateral-perfusion delay/defect more than one-half of the ischemic MCA territory in the late venous phase regardless of perfusion status at previous phases). The patient underwent conservative treatment and died of malignant infarction.

The SWI data were visually evaluated using a picture archiving and communicating system (PACS). A neuroradiologist (H.G.R., 19 years of experience) and a neurologist (S.B.L., 27 years of experience) who were blinded to all clinical data independently compared the prominent cortical and medullary veins between the affected hemisphere and the nonaffected hemisphere in terms of the degree of prominence using the following scoring system: 0, no prominent cortical or medullary vein in the affected hemisphere (Fig. 1); 1, mildly prominent cortical or medullary vein in the affected hemisphere (Fig. 2); 2, moderately prominent cortical or medullary vein (Fig. 3); and 3, very prominent cortical or medullary vein (Fig. 4). Two raters determined the final scores by consensus. Based on previous studies^30,31,33,35^, a collateral grading system was defined according to the scores of the prominent cortical and/or medullary veins as follows: good, intermediate, poor, and very poor (Table 1). The collateral-perfusion grading system of the multiphase MR angiography collateral map was defined by using the MR acute ischemic stroke collateral (MAC) scores as follows: 5, excellent; 4, good; 3, intermediate to good; 2, intermediate to poor; 1, poor; and 0, very poor^24,25^. We compared the collateral grades determined by SWI with the MAC scores of the multiphase MR angiography collateral map.

**Table 1:** Susceptibility-weighted imaging collateral grades.

Collateral grade	Description of collateral status
Good	No prominent cortical or medullary vein in the affected hemisphere (prominent cortical vein score 0 or prominent medullary vein score 0)
Intermediate	Mildly prominent cortical and/or medullary veins in the affected hemisphere (prominent cortical vein score 1 and/or prominent medullary vein score 1)
Poor	1. Moderately prominent cortical and/or medullary veins in the affected hemisphere (prominent cortical vein score 2 and/or prominent medullary vein score 2)
	2. Very prominent cortical or medullary veins in the affected hemisphere (prominent cortical vein score 3 or prominent medullary vein score 3)
Very poor	Very prominent cortical and medullary veins in the affected hemisphere (prominent cortical vein score 3 and prominent medullary vein score 3)

### Statistical analysis

The patient characteristics are expressed as the mean (SD), median (IQR) or number of participants (%). Differences in the distribution of patient characteristics among collateral grades were assessed using the chi-square test, Fisher's exact test, ANOVA and the Kruskal–Wallis test, as appropriate. The interrater reliability for collateral grading was measured by the Cohen weighted κ. Correlation between the collateral-perfusion grade of the multiphase MRA collateral map and SWI collateral grade was assessed using Spearman’s correlation. Multiple logistic regression analyses were conducted to identify independent predictors of favorable functional outcomes. The variables selected for the multivariable model were age, baseline NIHSS score, baseline DWI lesion volume, SWI collateral grade and reperfusion status. We included baseline predictors into multivariable models according to their *P* value (< 0.05) in the univariable model and significance in previous studies in multivariable models. To examine the linearity of the relationship between the collateral grades and functional outcomes, the likelihood ratio test for trends was used. Statistical analyses were performed using SAS, version 9.4 (SAS Institute Inc., Cary, NC). Statistical significance was indicated by *P* < 0.05.

## Results

### Participant characteristics

Of the 154 participants, 2 participants without SWI on baseline MRI were excluded. Hence, 152 participants were included in the current analysis. The mean age was 71 ± 13 years old (range = 34–92 years old), 64% of participants were men (98 men and 54 women), the median baseline NIHSS was 11 (interquartile range, 5–15), the median onset to door time was 58 min (interquartile range, 30–175 min), and the median onset to MRI time was 156 min (interquartile range, 92–284 min). Twenty-six participants (17.1%) presented with stenosis (> 50%) of the ICA or M1 segment of the MCA, 65 participants (42.8%) with occlusion of the M1 segment of the MCA, 17 participants (11.2%) with occlusion of the ICA, 38 participants (25%) with occlusion of the ICA and M1 segment of the MCA, and 6 participants (3.9%) with occlusion of the anterior cerebral artery accompanied by occlusion of the ICA and/or M1 segment of the MCA. Eleven participants (7.2%) underwent intravenous thrombolysis, 40 (26.3%) underwent intraarterial thrombectomy, 48 (31.6%) underwent intravenous thrombolysis and intraarterial thrombectomy, and 53 (34.9%) underwent conservative treatment. Sixty-eight participants (44.7%) achieved successful reperfusion.

### Relationship between the clinical data and SWI collateral grades

The interrater reliability of the SWI collateral grades (weighted κ = 0.93; 95% confidence interval (CI), 0.89 to 0.98) was almost perfect. The prominent vessel sign was demonstrated in 134 patients (88.2%). A good collateral grade was obtained in 18 participants (11.8%), an intermediate grade was obtained in 23 (15.1%), a poor grade was obtained in 33 (21.7%), and a very poor grade was obtained in 78 (51.3%). The demographic findings associated with each collateral grade are presented in Table 2. The absence of atrial fibrillation, a lower baseline NIHSS score and a smaller baseline DWI lesion volume were associated with higher collateral grades (all *P* < 0.001). The site of steno-occlusion and mode of treatment varied by the collateral grade (both *P* < 0.001). Occlusion rather than stenosis and a higher number of occluded arteries were related to a lower collateral grade and more aggressive treatment. The SWI collateral grade was positively correlated with the collateral-perfusion grade determined by the multiphase MR angiography collateral map (Spearman’s correlation coefficient = 0.45; *P* < 0.001) (Figs. 1, 2, 3, 4).

**Table 2:** Patient characteristics according to susceptibility-weighted imaging collateral grade.

Characteristics	Susceptibility-weighted imaging collateral grade				*P* value
	Very poor	Poor	Intermediate	Good	
No. of patients	78	33	23	18	
Men	16 (70)	15 (60)	24 (63)	16 (59)	0.28
Age (y)^†^	66 ± 12	75 ± 11	66 ± 13	71 ± 14	0.001
**Risk factors**					
Hypertension	44 (56.4)	20 (60.6)	12 (52.2)	8 (44.4)	0.71
Diabetes	21 (26.9)	8 (24.2)	6 (26.1)	5 (27.8)	0.99
Hyperlipidemia	18 (23.1)	10 (30.3)	4 (17.4)	2 (11.1)	0.41
Atrial fibrillation	37 (47.4)	11 (33.3)	3 (13.0)	1 (5.6)	< 0.001
Current smoker	16 (20.5)	3 (9.1)	7 (30.4)	5 (27.8)	0.17
Daily alcohol consumption	19 (24.4)	6 (18.2%)	6 (26.1%)	5 (27.8)	0.85
Previous TIA	5 (6.4)	1 (3.0)	2 (8.7)	1 (5.6)	0.84
Previous stroke	7 (9.0)	6 (18.2)	3 (13.0)	3 (16.7)	0.46
Previous ischemic heart disease	13 (16.7)	2 (6.1)	3 (13.0)	2 (11.1)	0.53
Peripheral artery disease	5 (6.4)	1 (3.0)	1 (4.3)	0 (0.0)	0.83
Baseline NIHSS score^‡^	12 (9–17)	13 (7–16)	4 (2–10)	5 (2–9)	< 0.001
Baseline DWI lesion volume^‡^	32 (11–83)	15 (5–26)	6 (1–13)	3 (1–7)	< 0.001
**Site of steno-occlusion**					< 0.001
ICA or M1 stenosis (> 50%)	1 (1.3)	2 (6.1)	13 (56.5)	10 (55.6)	
M1 occlusion	36 (46.2)	18 (54.5)	7 (30.4)	4 (22.2)	
ICA occlusion	9 (11.5)	3 (9.1)	3 (13.0)	2 (11.1)	
ICA and M1 occlusion	28 (35.9)	8 (24.2)	0 (0.0)	2 (11.1)	
ACA occlusion combined with ICA and/or M1 occlusion	4 (5.1)	2 (6.1)	0 (0.0)	0 (0.0)	
**Collateral grades of MRA collateral map**^§^					< 0.001
MAC 0	19 (24.4)	4 (12.1)	0 (0.0)	0 (0.0)	
MAC 1	19 (24.4)	3 (9.1)	1 (4.3)	1 (5.6)	
MAC 2	24 (30.8)	11 (33.3)	1 (4.3)	2 (11.1)	
MAC 3	13 (16.7)	8 (24.2)	2 (8.7)	3 (16.7)	
MAC 4	3 (3.8)	6 (18.2)	14 (60.9)	1 (5.6)	
MAC 5	0 (0.0)	1 (3.0)	5 (21.7)	11 (61.1)	
**Mode of treatment**					< 0.001
Conservative	15 (19.2)	12 (36.4)	12 (52.2)	14 (77.8)	
IVT only	5 (6.4)	3 (9.1)	1 (4.3)	2 (11.1)	
IAT only	24 (30.8)	10 (30.3)	5 (21.7)	1 (5.6)	
IVT followed by IAT	34 (43.6)	8 (24.2)	5 (21.7)	1 (5.6)	
**Functional outcome**^ǁ^					< 0.001
Favorable	38 (48.7)	13 (39.4)	21 (91.3)	14 (77.8)	
Unfavorable	40 (51.3)	20 (60.6)	2 (8.7)	4 (22.2)	

The mean overall patient age was 71 years ± 13 (standard deviation) (98 men and 54 women). Unless otherwise noted, the values are the number of patients, with percentages in parentheses.

*ACA* anterior cerebral artery, *IAT* intraarterial thrombectomy, *DWI* diffusion-weighted image, *ICA* internal carotid artery, *IVT* intravenous thrombolysis, *IQR* interquartile range, *M1* M1 segment of the middle cerebral artery, *MRA* magnetic resonance angiography, *N* number, *NIHSS* National Institutes of Health Stroke Scale, *TIA* transient ischemic attack.

^†^The data are the means ± standard deviation.

^‡^The data are the medians, with interquartile ranges in parentheses.

^§^The collateral grades of the MRA collateral map were the collateral grades estimated by the MR acute ischemic collateral (MAC) score of the multiphase MRA collateral map derived from dynamic contrast-enhanced MRA^24,25^.

^ǁ^A favorable functional outcome was defined as a modified Rankin scale (mRS) score of less than or equal to 2, and an unfavorable functional outcome was defined as an mRS score of greater than 2 at day 90.

### Multiple logistic regression analyses

The results of the multiple logistic regression analyses performed to identify independent predictors of favorable functional outcomes are presented in Table 3. In the univariable analysis, a younger age, a lower baseline NIHSS score, a smaller baseline DWI lesion volume, an intermediate collateral grade, a good collateral grade, and successful reperfusion were associated with favorable functional outcomes in the participants with acute ischemic stroke and included in the multivariable analysis. In the multivariable model, a younger age (adjusted odds ratio (aOR), 0.42; 95% CI 0.34 to 0.77; P < 0.001), a lower baseline NIHSS score (aOR 0.90; 95% CI 0.82 to 0.98; *P* = 0.02), a smaller baseline DWI lesion volume (aOR 0.83; 95% CI 0.73 to 0.96; *P* = 0.01), an intermediate collateral grade (aOR 9.49; 95% CI 1.36 to 66.38; *P* = 0.02), a good collateral grade (aOR 6.22; 95% CI 1.16 to 33.24; *P* = 0.03), and successful reperfusion (aOR 5.84; 95% CI 2.08 to 16.42; *P* = 0.001) were independently associated with a favorable functional outcomes. There was a linear negative association between the collateral grade and functional outcome (*P* = 0.008) after adjusting for covariates.

**Table 3:** Results of logistic regression analysis: independent predictors of favorable functional outcome.

Predictor	Univariable model		Multivariable model	
	Odds ratio	*P* Value	Odds ratio	*P* value
Age, per 10-year increase	0.51 (0.37, 0.71)	< 0.001	0.42 (0.34, 0.77)	< 0.001
Baseline NIHSS score	0.84 (0.78, 0.89)	< 0.001	0.90 (0.82, 0.98)	0.02
Baseline DWI lesion volume	0.79 (0.71, 0.88)	< 0.001	0.83 (0.73, 0.96)	0.01
**Collateral grade**				
0 (Very poor)	Reference		Reference	
1 (Poor)	0.68 (0.30, 1.57)	0.37	1.21 (0.38, 3.83)	0.75
2 (Intermediate)	11.05 (2.42, 50.37)	0.002	9.49 (1.36, 66.38)	0.02
3 (Good)	3.68 (1.11, 12.19)	0.03	6.22 (1.16, 33.24)	0.03
*P* for trend^†^		< 0.001		0.008
Successful reperfusion^‡^	2.58 (1.32, 5.04)	0.01	5.84 (2.08, 16.42)	0.001

The data in parentheses are the 95% confidence intervals.

A favorable functional outcome was defined as a modified Rankin scale (mRS) score of less than or equal to 2, and an unfavorable functional outcome was defined as a mRS score of greater than 2 at day 90.

*DWI* diffusion-weighted imaging, *NIHSS* National Institutes of Health Stroke scale.

^†^*P* values for the linearity of the relationship between the collateral grades and functional outcomes.

^‡^Successful reperfusion was defined as a mTICI score of 2b to 3 after intraarterial thrombectomy.

## Discussion

Our study showed a positive correlation between the SWI collateral grade and collateral-perfusion grade determined by the multiphase MR angiography collateral map (*P* < 0.001) and a linear negative association between the SWI collateral grades and functional outcomes (*P* = 0.008). We demonstrated that the significant predictors of a favorable functional outcome in acute ischemic stroke due to occlusion or stenosis of the unilateral ICA and/or M1 segment of the MCA within 8 h of symptom onset were a younger age, lower baseline NIHSS scores, and smaller baseline DWI lesion volume, an intermediate and good SWI collateral grade and reperfusion.

There are several studies on the value of the prominent vessel sign on SWI for collateral estimation and prognosis prediction, with results differing by the conditions of the subjects and study methods^30–32,34,38^. In Verma’s study comparing the conventional angiography of 33 patients with acute ischemic stroke due to M1-segment occlusion, more pronounced prominent cortical veins on SWI correlated with poor collateralization on conventional angiography, while less pronounced prominent cortical veins correlated with good collateralization, and the prominence grade of the prominent cortical veins and baseline NIHSS scores were lower in the group with good collateralization than in the group with poor collateralization^30^. Sum et al., Chen et al. and Li et al. showed that prominent cortical veins are associated with early neurologic deterioration and/or unfavorable prognoses^31,32,34^. In contrast, in Park’s study comparing hyperintense vessel signs on FLAIR and vessels on postcontrast time-of-flight MR angiography in 80 patients with acute ischemic stroke due to occlusion of the ICA or proximal MCA who underwent MRI within 3 days from stroke onset, more extensive prominent cortical veins on SWI were associated with lower baseline NIHSS scores, smaller baseline DWI lesion volumes, and better collateral flow, which are expected factors of favorable prognoses^37^. Park et al. assessed the extent of prominent cortical veins by the modified Alberta Stroke Program Early Computed Tomography Score (ASPECTS) system, but Verma et al. assessed the degree of prominence. Without considering the degree of prominence, a mildly prominent cortical vein can be associated with good collateral status, although its extensiveness is similar to that found in Park’s study which included subacute-stage infarction. Additionally, as shown in Fig. 1 of Park’s study, cerebral edema can obscure the prominent cortical vein, especially in progressed large infarction, which is an important pitfall to be aware of when interpreting collateral status with SWI. Furthermore, Park et al. compared their SWI collateral grades with the hyperintense vessel sign on FLAIR and postcontrast time-of-flight MR angiography. Collateral estimation using imaging methods without time resolution, such as single-phase computed tomography angiography, FLAIR, and postcontrast time-of-flight MR angiography, is associated with a higher risk of mislabeled pial arterial filling than collateral estimation using imaging methods with time resolution, such as multiphase computed tomography angiography, dynamic contrast-enhanced MR angiography, and dynamic susceptibility contrast-enhanced MR perfusion^22–24,39–44^. The use of imaging methods without time resolution leads to the over- or under-estimation of collateral status, especially in patients with late time windows over a day. The multiphase MR angiography collateral map derived from dynamic contrast-enhanced MR angiography is a precise method used for collateral estimation that can provide detailed information about tissue-level, brain parenchymal collateral perfusion and cortical artery information according to an individual’s hemodynamics^24^. Therefore, it is an optimal method for verifying the value of prominent vessel signs on SWI for collateral estimation. Our study directly showed the association between the degree of prominence of the prominent vessel sign and collateral status estimated with a multiphase MR angiography collateral map (Table 2, Figs. 1, 2, 3, 4)^24,25^.

Mucke et al. showed that prominent medullary veins on SWI were associated with higher NIHSS scores at admission and discharge and higher mRS scores at discharge^33^. Jing et al. showed that prominent cortical and medullary veins on SWI were associated with higher baseline NIHSS scores, larger baseline infarct volumes, and larger infarct growth at 7 days than prominent cortical vein only^35^. A quantitative study of susceptibility using SWI showed that the presence of a prominent cortical vein corresponded to reduced levels of oxygen saturation^36^. On the basis of these studies, we developed an SWI collateral grading system by introducing the concepts of prominent cortical and medullary veins and the degree of the prominence of these veins (Table 1). We expected that this 4-collateral grade system of SWI could yield more sophisticated collateral and perfusion information on the whole brain than other noncontrast imaging method for collateral estimation, such as FLAIR and time-of-flight MR angiography. This study revealed a significant correlation between the SWI collateral grading system and the 6-collateral grading system of the multiphase MR angiography collateral map and a linear negative association between the SWI collateral grading system and the 6-score mRS on day 90. In Xu’s study comparing hyperintense vessel signs on FLAIR in 56 patients with large cerebral artery occlusion, prominent medullary veins were associated with good collateral flow^45^. The authors did not consider the degree of the prominence either and used the hyperintense vessel sign on FLAIR for collateral estimation comparisons, and this method has the same limitation as mentioned in the above paragraph. Our study also showed that more prominent cortical and medullary veins are associated with the presence of atrial fibrillation. This finding is consistent with the results of previous studies that showed that stroke due to atherosclerotic steno-occlusive disease was associated with better collateral status than cardioembolic stroke^46–48^. Abrupt development of ischemic stroke by cardioembolic occlusion is thought to be associated with a paucity of previously developed collaterals leading to more severe hypoperfusion and an increase in the oxygen extraction fraction in the acute period^49,50^. In contrast, patients with atherosclerotic steno-occlusive disease may maximize collateral recruitment due to its chronic and slowly progressive nature^51^. Acute ischemic stroke due to large vessel occlusion is commonly due to cardioembolism or atherosclerotic steno-occlusive disease. Identification of these subtypes is important to establish an endovascular treatment strategy that enhances timely recanalization, but is frequently impossible before the procedure. Prominent cortical and/or medullary veins on SWI can be an indicator to neurointerventionists that the cause of large vessel occlusion is more likely cardioembolism rather than atherosclerotic steno-occlusive disease.

Recently, developed collateral imaging methods, such as multiphase CT angiography^22^ and the multiphase MR angiography collateral map^24^, despite their usefulness in treatment decision making, are not suitable for patients for whom iodine- or gadolinium contrast material is not recommended^28^. FLAIR and arterial spin labeling have been introduced as non-contrast imaging methods for collateral estimation^41,52,53^. Studies have shown hyperintense leptomeningeal vessels on FLAIR could indicate slow arterial flow in acute ischemic strokes and represent collateral circulation, but vessel signals at a particular time point cannot provide precise collateral information, as we mentioned already^40,43^. Arterial spin labeling is representative noncontrast perfusion imaging, and arterial transit artifacts, which are bright arterial spin labeling signals produced by delayed flow, can be used to estimate collateral circulation. Arterial spin labeling is a promising method but has several disadvantages, including lengthy acquisition time, susceptibility to motion and denture artifacts, (especially in elderly patients with acute ischemic stroke), and lack of a collateral grading method^40,43,44^. SWI is an essential sequence with high resolution in MR protocols for hemorrhage detection in patients with acute ischemic stroke and is easily acquired with any MR scanner within 100 s. Our study suggested a practical collateral grading system for SWI and showed the possibility of performing collateral imaging without contrast material or additional acquisition time. Additionally, the susceptibility vessel sign defined as a hypointense signal that exceeds the diameter of the contralateral artery is highly specific for the location of the occluded artery and represents a recanalization prediction after IVT^54^. The susceptibility vessel sign is also helpful for aiding neurointerventionists in planning the IAT treatment.

One limitation of our study is that the prominent cortical and medullary veins were assessed visually. However, the interrater reliability was almost perfect, and the collateral grading system can be applied without further postprocessing. We expect that the development of an automatic calculation method using a susceptibility threshold to indicate salvageable brains in patients with acute ischemic stroke can provide more precise information on collaterals and the penumbra.

In conclusion, collateral estimation using the prominent vessel sign on SWI is clinically reliable, as it has prognostic value. It is particularly useful in patients for whom contrast material is not recommended.
